# Integrated proteomics, genomics, metabolomics approaches reveal oxalic acid as pathogenicity factor in *Tilletia indica* inciting Karnal bunt disease of wheat

**DOI:** 10.1038/s41598-018-26257-z

**Published:** 2018-05-18

**Authors:** Vishakha Pandey, Manoj Singh, Dinesh Pandey, Anil Kumar

**Affiliations:** Department of Molecular biology and Genetic Engineering, G.B. Pant University of Agriculture and Technology, Pantnagar, Uttarakhand India

## Abstract

*Tilletia indica* incites Karnal bunt (KB) disease in wheat. To date, no KB resistant wheat cultivar could be developed due to non-availability of potential biomarkers related to pathogenicity/virulence for screening of resistant wheat genotypes. The present study was carried out to compare the proteomes of *T. indica* highly (TiK) and low (TiP) virulent isolates. Twenty one protein spots consistently observed as up-regulated/differential in the TiK proteome were selected for identification by MALDI-TOF/TOF. Identified sequences showed homology with fungal proteins playing essential role in plant infection and pathogen survival, including stress response, adhesion, fungal penetration, invasion, colonization, degradation of host cell wall, signal transduction pathway. These results were integrated with *T. indica* genome sequence for identification of homologs of candidate pathogenicity/virulence related proteins. Protein identified in TiK isolate as malate dehydrogenase that converts malate to oxaloacetate which is precursor of oxalic acid. Oxalic acid is key pathogenicity factor in phytopathogenic fungi. These results were validated by GC-MS based metabolic profiling of *T. indica* isolates indicating that oxalic acid was exclusively identified in TiK isolate. Thus, integrated omics approaches leads to identification of pathogenicity/virulence factor(s) that would provide insights into pathogenic mechanisms of fungi and aid in devising effective disease management strategies.

## Introduction

*Tilletia indica* is a smut fungus that incites Karnal bunt (KB), an economically important disease of wheat. It was first reported from Karnal city of India in 1931^1^, now it is widespread in many countries, including Afganistan, Pakistan, Iran, Iraq, South Africa, United States and Mexico **(**http://www.nda.agric.za/docs/GenPub/karnalbunt.htm**)**. A floret infecting disease, KB partially converts the kernels into sori filled with fetid teliospores and reduces the crop yield and seed quality. Wheat products prepared from wheat grains containing 1% or more bunted kernels becomes inedible because of offensive fishy smell emitted by teliospores. The wheat lot that exceeds 3% infected seeds becomes unfit for human consumption, downgrading the high quality wheat grains to animal feed^2^. This causes large financial losses to the wheat producing countries. The disease also affects the international trade as many countries pose quarantine barrier against *T. indica* by virtue of which wheat exporters face the restriction in the movement of wheat consignments to countries where KB pathogen is not yet reported^3^. Now, more than 70 countries have placed *T*. *indica* as a quarantine pest^4^. Further, some wheat importing countries have placed strict quarantine regulations against *T. indica* due to which they have to bear indirect costs for implementation of quarantine measures.

The fungus *T*. *indica* being heterothallic in nature, forms haploid secondary sporidia. The compatible sporidia come in contact, hybridize and result in infection. Thus, *T. indica* shows high level of diversity between as well as within the species. Due to high genetic variability among the *T. indica* isolates, they exhibit varying degree of virulence. Different isolates of *T. indica* exhibit differential virulence levels. Consequently, *T. indica* isolates exhibiting contrasting virulence behaviour may also differ in the expression of pathogenicity proteins/virulence factors. Plant pathogenic fungi employ several pathogenicity/virulence related proteins to infect their host plants. They are classified as components of the signal transduction pathways, enzymes for degrading host defenses, transporters to defend the fungus against host defences, toxins effective against host defences and penetration effectors viz. hydrophobins, glycerol and melanin^5^. The pathogenicity/virulence related proteins may serve as potential biomarkers for screening of resistant wheat genotypes and diagnosis of KB pathogen.

In this context, it is pertinent to differentiate the *T. indica* isolates based on the degree of virulence/aggressiveness which in turn can be employed for the plant breeding programme. In the present study, the virulence of different *T. indica* isolates was compared on the basis of their pathogenic variability tested on a set of host differentials of *Triticum aestivum*, in order to select isolates exhibiting contrasting virulence behaviour for comparative proteomic analysis of mycelial proteins for identification of pathogenicity or virulence related proteins.

In the field of fungal proteomics, 2-DE coupled to tandem mass spectrometry has been extensively used for studying fungal plant pathogens with respect to their proteome maps, pathogenicity proteins and virulence factors^6–8^. With this view, the present study was aimed to carry out the comparative proteomic analysis of mycelial proteins from *T. indica* isolates exhibiting varied virulence behaviour. The results obtained by comprehensive proteomic analysis would be further integrated with the *de novo* genome sequence generated employing the hybrid approach of Illumina HiSeq. 2000 and PacBio single molecule real time (SMRT) sequencing technology^9^. The identified homologs of candidate pathogenicity/virulence related proteins from *T. indica* genome were further subjected to sequence and structure based functional annotation. The metabolic/gene regulatory networks involved in conferring virulence behaviour can also be analysed by metabolite profiling through metabolomics. Most of the metabolomic studies conducted till date monitor the metabolomic changes in host plant upon infection by the fungal pathogens^10,11^. Certainly, there is no information available regarding the metabolomic study on this important plant pathogenic fungus. To monitor and identify the difference between the *T. indica* isolates exhibiting varying virulence levels, metabolomes were carried out that would further complement our results obtained by proteomics and genomics studies.

The prime objective of the present study is to integrate the comparative omics data of *T. indica* isolates showing varied virulence behaviour and the interpretation of the results would facilitate the identification of pathogenicity or virulence related proteins of this economically important fungus.

## Materials and Methods

### Fungal isolates, plant material and growth conditions

Karnal bunt infected wheat grain samples were collected from Indian Institute of Wheat and Barley Research (IIWR), Karnal. Cultures of *T. indica* isolates were grown from a single teliospore using technique described by Warham^12^ and further multiplied on modified potato dextrose agar (PDA) in biological oxygen demand (BOD) incubator under alternating dark and light conditions at 20 ± 2 °C for 15–20 days. Fungal isolates were maintained on both liquid and solid modified potato dextrose medium. Ten wheat varieties showing differential disease response to *T. indica* isolates were generously provided by Dr. M.S. Saharan from IIWR Karnal. Wheat seeds were planted in Crop Research Centre, G.B. Pant University of Agriculture and Technology, Pantnagar for pathogenicity testing studies.

### Pathogenic Variability Test

Pathogenic variability of ten *T. indica* isolates was evaluated on a set of ten host differentials of *Triticum aestivum* for two consecutive years. All differentials of wheat were sown in a meter row with 25 cm line – to – line spacing in mid-November (the normal wheat sowing time). Need based watering and doses of fertilizer were applied as recommended by Tandon and Sethi^13^ and each host was maintained in triplicates About 10^4^/ml sporidial suspension from each isolate was used to inoculate the five tillers of each host differential at boot leaf stage^14^ using hypodermic syringe according to the procedure described by Aujla *et al*.^15^. High-humidity was maintained by using a mist sprayer for at least 4 hours a day to ensure infection. Un-inoculated ear heads for each differential were used as negative control. Bunted ear heads were harvested and manually threshed after maturity. Infected grains were scanned visually and categorized into five different grades of infection based on the conversion of the endosperm region into teliospores sooty mass. Percent coefficient of infection was calculated following Aujla *et al*.^16^. Based on percent coefficient of infection (CI) and percent incidence for two successive crop seasons, *T. indica* isolates were categorized as highly aggressive, moderately aggressive and low aggressive. CI data was subjected to two way analysis of variance (ANOVA) using STPR 2.0 software.

### Harvesting of mycelium

*T. indica* isolates growing in modified potato dextrose broth (PDB) were harvested at different time intervals, 14^th^, 21^st^, 30^th^ and 40^th^ days. Liquid culture containing fungal mycelia mat was filtered using folded muslin cloth. Washing was done first with phosphate buffer saline (PBS) (0.05 M, pH 7.2), then with sterilized distilled water. Wet fungal mycelium was lyophilized for 6 hours. Dried mycelia biomass from *T. indica* cultures at different time intervals were used for plotting growth curve and stored at −80 °C until used for protein isolation.

### Extraction of mycelial proteins

Protein extracts from *T. indica* isolates showing differential virulence behaviour were obtained from lyophilized mycelia (0.5 g) using PBS (pH 7.2) as extraction buffer. Extracted mycelia proteins were precipitated by 10% TCA in acetone and final pellet was dissolved in a solubilisation buffer (8 M urea; 2 M thiourea; 2% [w/v] CHAPS; 50 mM DTT). Extracted protein from each isolate was quantified by Bradford assay^17^ using bovine serum albumin as standard (BSA) as standard.

### Two – dimensional gel electrophoresis (2–DE) and image analysis

For 2–DE, 125 μg of mycelial proteins was dissolved and rehydrated in 125 μL of IEF rehydration buffer (8 M urea; 2 M thiourea; 2% [w/v] CHAPS; 0.2% [v/v] IPG buffer 3–10; 50 mM DTT; 0.01% [w/v] bromophenol blue) for 16 h following Bio-Rad protocol. IEF was performed on Bio-Rad Protean IEF Cell system according to Fragner *et al*.^18^ with slight modifications. IPG focused strips were reduced and alkylated using equilibriation buffer I (0.375 M Tris (pH 8.8), 6 M urea, 20% glycerol, 10% SDS and 130 mM DDT) and equilibriation buffer II (0.375 M Tris (pH 8.8), 6 M urea, 20% glycerol, 10% SDS and 130 mM Iodoacetamide). Second-dimension was conducted on 12% acrylamide gels at 100 V (Mini – PROTEAN Tetra Cell, Bio-Rad). The gel image was acquired by alphaimager gel documentation system (Proteinsimple, California, USA). Gel analysis was performed using IMP7 software. Only consistently overexpressed and differentially expressed spots in three biological replicates were chosen for identification by MALDI- TOF/TOF.

### Tandem Mass Spectrometry and Database Searching

For in gel-digestion of proteins, the protein spots were manually excised from CBB stained 2-DE gels and suspended in 10% glacial acetic acid. Excised spots were destained with 50 mM NH_4_HCO_3_ in 50% (v/v) methanol at 40 °C for 1 hour. After being completely dried in vacuum centrifuge, gel particles were digested with 5 ng/µl of trypsin at 37 °C for 16 h. The digested peptides were extracted using 0.1% trifluoroacetic acid (TFA) in 50% acetonitrile. Peptides were resuspended in α-cyano-4-hydroxycinnamic acid in 50% acetonitrile containing 0.1% TFA. Mass spectra was obtained using tandem mass spectrometry (ULTRAFLEX III TOF/TOF, Bruker Daltonics). Mass spectrometry spectra were acquired with 1600 laser shots per spectrum and MS/MS spectra with 2500 laser shots per fragmentation spectrum. 10 strongest peaks from MS spectra were chosen as the precursor ions to attain the MS/MS fragmentation spectra. The Flex analysis software 3.0 (Bruker Daltonics) was used for spectra analysis and generation of peak list files. The peak list files were searched in NCBI nonredundant (NR) database (http://www.ncbi.nlm.nih.gov), version 20160114, 79354501 sequences and 28992349963 residues for “Fungi”) through the MASCOT search engine **(**http://www.matrixscience.com). Search parameters were kept to taxonomy, Fungi; proteolytic enzyme, trypsin; max missed cleavages, 1; fixed modifications, carbamidomethyl (C); variable modifications, oxidation (M); peptide mass tolerance, 50 ppm; fragment mass tolerance, 2 Da. Functional annotation of identified proteins was performed by Uniprot database (www.uniprot.org/). Identified proteins were grouped into distinct categories through COG and GO pathway analysis at NCBI. Subcellular localization of identified proteins was carried out by TargetP^19^.

### Identification of homologs of pathogenicity/virulence related proteins from *T. indica* genome sequence data

Genome of *T. indica* TiK isolate sequenced^9^ was used for identification of homologs of candidate pathogenicity/virulence related proteins using BLASTP search. Identified putative pathogenicity/virulence related proteins from *T. indica* genome were subjected to further sequence and structure based functional annotation.

### Sequence and structure based functional annotation of homologs of pathogenicity/virulence related proteins

Identified putative pathogenicity/virulence related proteins homologs from TiK genome were extensively analyzed for the functions by InterProScan^20^ and NCBI conserved domain database (CDD)^21^. Other databases such as ScanProsite^22^, SMART^23^, PANTHER^24^ and CATH^25^ were also used for sequence based functional analysis of proteins. Three dimensional structures of identified putative pathogenicity/virulence related proteins were predicted using RaptorX software^26^. Predicted protein models were validated by various bioinformatics tools such as RAMPAGE^27^, ProQ server^28^, DALI server^29^ and ProFunc^30^.

### Protein-Protein interaction network

Protein sequences of putative pathogenicity/virulence related genes retrieved from genome sequence of *T. indica* TiK isolate were used to generate Protein-Protein interaction (PPI) network by STRING (Search Tool for the Retrieval of Interacting Genes) database version 10.5^31^ taking *Saccharomyces cerevisiae* as a model system. The obtained PPI network was further visualized using Cytoscape version 3.5.1^32^.

### Gas Chromatography – Mass Spectrometry (GC-MS)

GC-MS was performed for metabolic profiling of *T. indica* isolates showing extreme virulence behaviour, TiK and TiP, at Advanced Instrumentation Research Facility (AIRF), JNU New Delhi. Each sample (20 mg) was extracted using 1 ml of 70% methanol. Extract was sonicated and centrifuged at 5,000 rpm for 10 minutes. Supernatant was collected and filtered through a 0.45 mm filter. Sample (100 µl) was transferred into a GC vial and dried with a nitrogen gas flow at 60 °C for 5 min. For derivatization, each sample (100 µl) was oximated with 50 µl methoxyamine hydrochloride in pyridine. After incubation at 30 °C for 90 min, samples were silylated with 25 µl of N-methyl-N-trimethylsilyl trifluoroacetamide (MSTFA) at 37 °C for 60 min. GC- MS was performed according to Oh, *et al*.^33^.

## Results

### Differential disease scoring on a set of host differentials of *Triticum aestivum* suggests varied virulence behaviour of *T. indica* isolates

**Figure 1:**
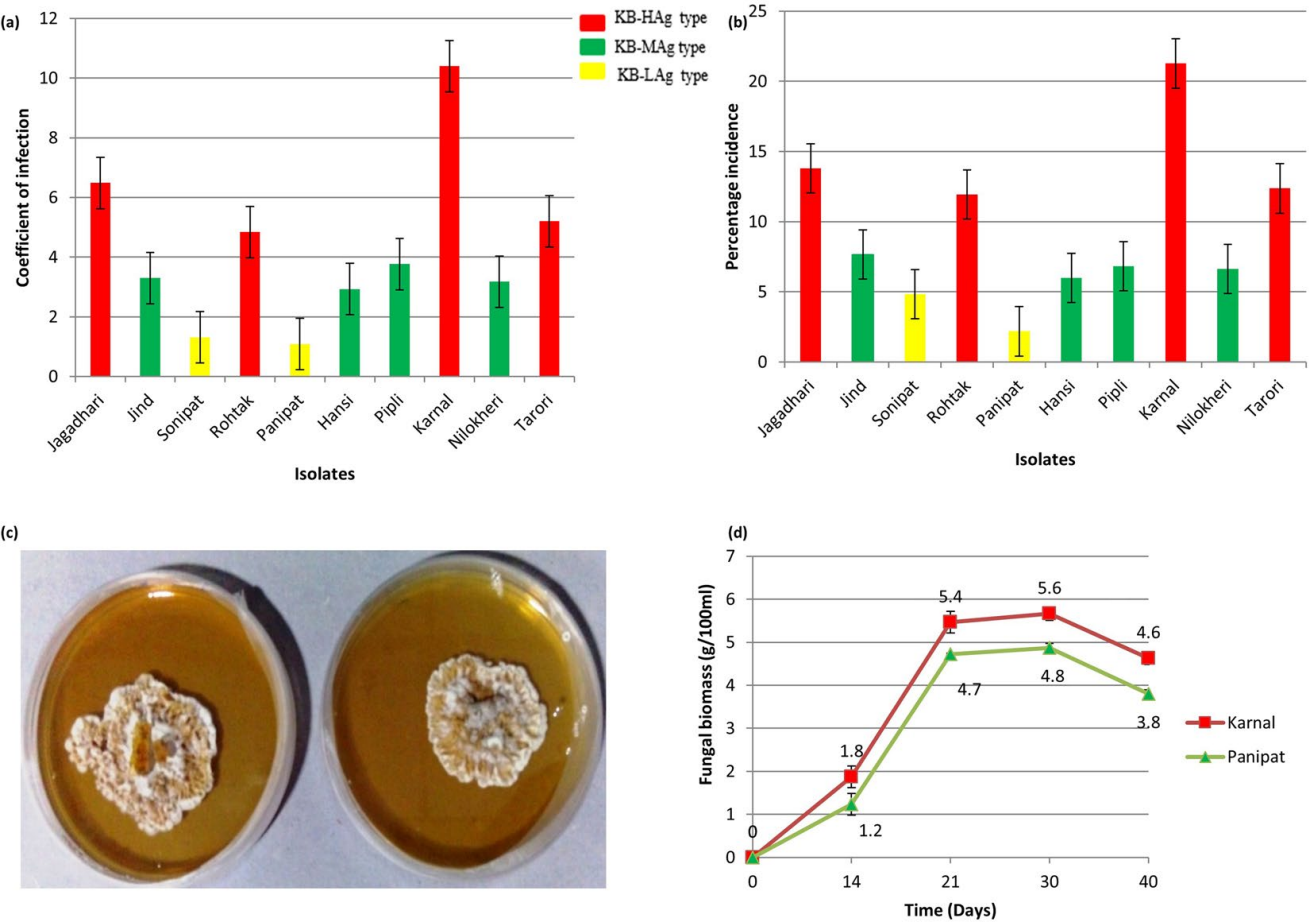
Comparison of virulence of ten *T. indica* isolates based on differential disease scoring on a set of ten host differentials of *Triticum aestivum* in terms of (**a**) Coefficient of infection; (**b**) Percentage incidence. Each value represent the average of three biological replicates; (**c**) the growth of *T. indicia* highly virulent TiK and low virulent TiP after 21 days of culture on PDA; (**d**) Growth kinetics of TiK and TiP isolate in terms of total biomass production (g/100 ml) at different time intervals.

Pathogenicity of ten isolates of *T. indica* was evaluated on a set of ten host differentials of wheat for two successive years (Year 2013–14 and 2014–15). The average coefficient of infection and percent incidence calculated from the field experiment is presented in Fig. 1a,b. The pathogenic variability among the isolates is indicated by host–pathogen interaction on differential hosts. The *T. indica* isolates can be categorized into three distinct aggressive types, namely Karnal Bunt- High Aggressive type (KB-HAg), Karnal Bunt- Moderate Aggressive type (KB-MAg) and Karnal Bunt- Low Aggressive type (KB-LAg). Four isolates belonged to KB-HAg type, four isolates to KB-MAg type and remaining two isolates to KB-LAg type. The KB-HAg comprised the isolates from Karnal (TiK), Jagadhari (TiJg), Rohtak (TiR) and Tarori (TiT). Two isolates of the pathogen that belonged to KB-Lag type were represented from Panipat (TiP) and Sonipat (TiS). The KB-MAg type of *T. indica* was from Jind (TiJ), Hansi (TiH), Pipli (TiPp) and Nilokheri (TiN).

The highest per cent incidence of disease was produced by TiK isolate (21.2%), followed by TiJg (13.8%) and TiT isolate (12.3%). The TiP isolate showed the lowest per cent incidence of disease i.e. 2.1% (Fig. 1b). The pathogenicity variability test also suggested that the TiK and TiP isolate as the most aggressive and the least aggressive type as they exhibited the highest (10.4) and the lowest (1.1) coefficient of infection, respectively on most of the host differentials as compared to other isolates (Fig. 1a). Thus, these isolates exhibiting differential virulence behaviour are used in the present investigation for comparative proteomic analysis for identification of pathogenicity/virulence related proteins. ANOVA exhibited the significant differences in disease severity among the isolates, wheat genotypes and their interactions. The means square for the genotypes was relatively lower (56.8) than for the isolates (353.3). The mean square for the genotype X isolate interaction was very high (59.9) (Table 1).

**Table 1:** Analysis of variance for Karnal bunt reaction of 10 isolates of *T. indica* on a set of 10 host differential of *T. aestivum*.

Source of variation	Pathogenic variability			
	Sum of Squares	Degree of Freedom	Mean Square	F value
Isolates (I)	3180.5	9	353.3	54.1**
Host genotypes (H)	511.3	9	56.8	8.7**
IXH	4856.8	81	59.9	9.1**
Residual	1291.9	198	6.5	
Total	9840.7	299		

### Comparative proteomic analysis of *T. indica* isolates exhibiting varied virulence behaviour

In proteomic research, 2-DE coupled to MS serve as powerful tools for identification of isolate-specific expression of pathogenicity or virulence related proteins in various plant pathogenic fungi such as *Botrytis cinerea*, *Verticillium dahliae*, *Fusarium oxysporum* f. sp. *conglutinans*^6–8,34^.

The growth rates of TiK and TiP isolate cultured on PDA and PDB medium were compared from 1 to 40 days by calculating the fungal biomass (g/100 ml of culture). The mycelium of both TiK and TiP increased exponentially (logarithmic growth phase) up to 21 days followed by decrease in the rate of growth (stationary growth phase) (Fig. 1d). The fungal biomass of TiK isolate at lag phase (14^th^ day) was approximately 1.8 g/100 ml of culture which increased upto 5.4 g/100 ml at 21^st^ day of growth (exponential phase) (Fig. 1d). In TiP isolate, fungal biomass 1.2 g/100 ml at 14^th^ day which rose to 4.7 g/100 ml of culture at 21^st^ day growth cycle. After 21^st^ day, decrease in growth in terms of dry weight of mycelia was observed in both the isolates (Fig. 1d). It is due to exhaustion of available nutrients in media. As shown in Fig. 1d, highly aggressive TiK isolate grew at a more rapid rate than TiP isolate. Both TiK and TiP isolates reach exponential phase of growth at 21^st^ day of growth. Thus, 21 day old cultures of *T. indica* isolates, TiK and TiP, were selected for comparative proteomic studies.

**Figure 2:**
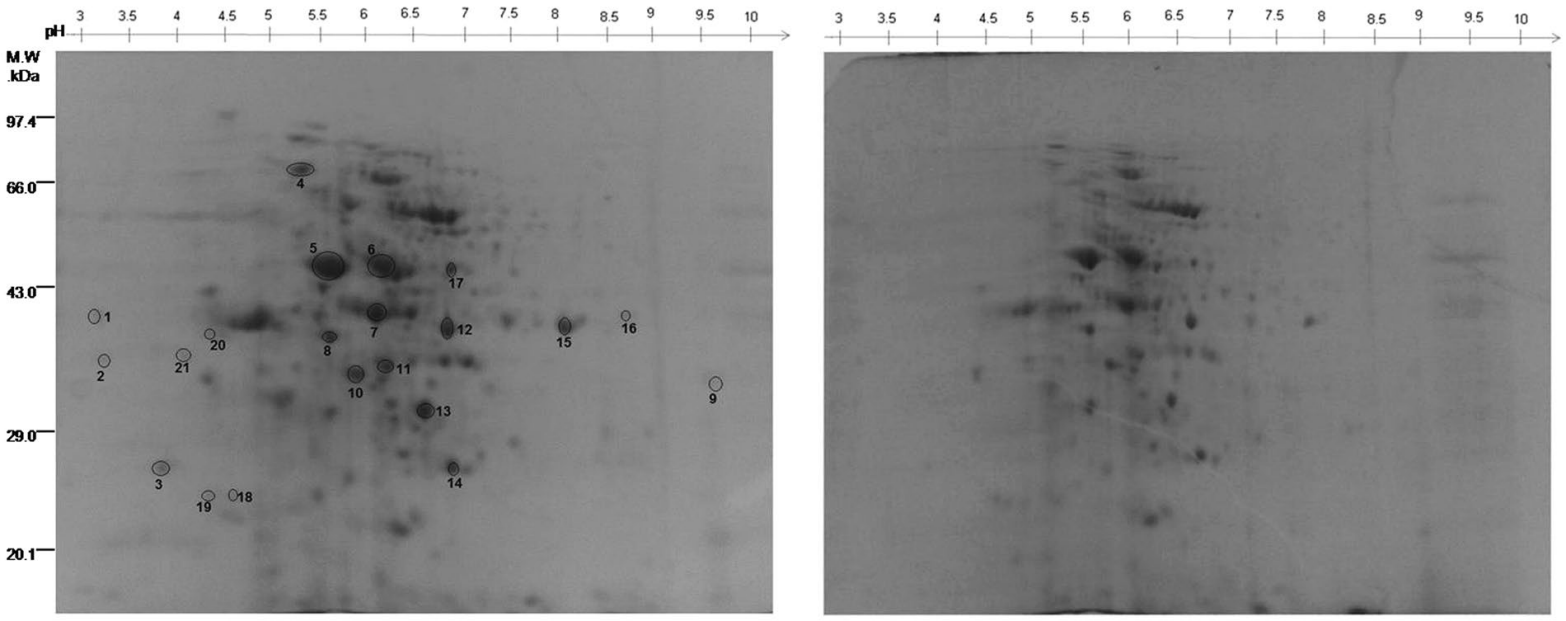
2-DE gel of proteins extracted from mycelia of *T. indica* isolates (at 21 days of growth) showing contrasting virulence behaviour (**a**) 2-DE gel image of TiK, highly virulent isolate (**b**) TiP, low virulent isolate. Proteins spots exhibiting higher or differential expression in TiK isolate are encircled. Representative 2-DE gels are shown in Supplementary Fig. S1.

A proteome map (pH 3–10) of protein samples from the mycelia of two *T. indica* isolates, TiK and TiP, varying in their aggressiveness or virulence levels was obtained with comparative proteomic analysis of high quality 2-DE gels (Fig. 2; Supplementary Fig. S1). By comparing the proteome profiles from these two isolates, various qualitative variations between protein spots were revealed. Only those spots that were consistently expressed in three replicate gels of highly virulent TiK isolate were considered for further analysis. Twenty one protein spots that exhibited more than a 1.5 fold change were statistically significant (p < 0.05) and selected for tandem mass spectrometry analysis. Among them, proteins corresponding to spots 3–15 and 17 showed higher expression in TiK isolate and proteins in spots 1, 2, 16, 18, 19, 20, 21 were found to be differentially expressed with respect to TiP isolate (Fig. 2). The proteins of such spots were subjected to MALDI-MS/MS analysis.

All the differentially expressed or up- regulated proteins were identified by MASCOT search as protein with known functions, except the protein from spot 3, 14 and 21, that were identified as a hypothetical protein. The calculated molecular mass of the identified proteins ranged from approximately 13 to 395 kDa. The protein spots identified are indicated by circles and numbers on the gels (Fig. 2). The putative protein identity, protein score and number of matched peptides are given in Table 2.

**Table 2:** Identification of differentially expressed and up-regulated proteins in TiK, the highly virulent *T. indica* isolate by MALDI-MS/MS and MASCOT.

Spot No.^a^	Accession ID^b^	Putative protein identity	Organism	Theo. pI/Mr^c^	Exper. pI/Mr^d^	Protein Score^e^	Peptide no.^f^
1	587674393	ATPase protein	*Fusarium oxysporum*	5.93/83.36	3.25/40.46	91	12
2	154269657	Glycosyl hydrolase family 45	*Beauveria bassiana*	5.37/44.85	3.80/38.67	80	6
3	924814796	Hypothetical protein	*Malassezia pachydermatis*	5.53/63.81	3.96/25.98	97	9
4	576987972	Hsp70 protein	*Rhizoctonia solani*	5.18/72.24	5.50/73.00	87	10
5	768764200	Rga2p	*Saccharomyces cerevisiae*	7.92/1144.45	6.00/44.24	98	8
6	757378465	STE/STE7 protein kinase	*Cryptococcus gattii*	7.64/64.80	6.50/44.24	88	15
7	448529472	Fructose-bisphosphate aldolase	*Candida orthopsilosis*	5.53/39.83	6.50/40.50	135	5
8	630344533	Dyenin heavy chain	*Gloeophyllum trabeum*	5.46/394.35	7.92/39.47	90	23
9	671695432	Malate dehydrogenase	*Lichtheimia ramose*	6.24/59.55	6.00/36.86	80	11
10	671695432	Malate dehydrogenase	*Lichtheimia ramose*	6.24/59.42	6.06/39.23	80	10
11	302894299	Dyenin heavy chain	*Cylindrobasidium torrendii*	5.42/393.86	6.58/39.3		20
12	758984299	Malate dehydrogenase	*Ustilago maydis*	6.92/34.13	7.25/40.32	253	6
13	939400439	β-1,3-Glucanosyltransferase	*Ceraceosorus bombacis*	9.48/96.84	6.93/31.34	107	5
14	953439809	hypothetical protein	*Bipolaris victoriae*	10.52/13.37	7.28/26.00	81	9
15	808374308	Glyceraldehyde-3-phosphate dehydrogenase	*Pseudozyma hubeiensis*	9.11/42.08	8.45/40.43	131	7
16	443897433	Glyceraldehyde 3-phosphate dehydrogenase	*Pseudozyma Antarctica*	7.66/36.05	9.00/40.45	82	5
17	813217281	Malate dehydrogenase	*Saitoella complicata*	4.27/40.84	7.20/44.24	130	5
18	821061148	Putative protein kinase	*Phaeomoniella chlamydospora*	8.75/57.38	4.92/24.55	123	8
19	630968700	Glycine decarboxylase	*Anthracocystis flocculosa*	9.08/49.86	4.90/23.20	74	10
20	647401639	CorA-like Mg2+ transporter protein	*Rhodosporidium toruloides*	6.47/88.58	4.63/39.82	93	7
21	759210621	hypothetical protein	*Exophiala mesophila*	5.50/160.56	4.32/39.25	86	11

^a^Designated Spot numbers 1–21 correspond to the 2-DE gel (Fig. 2); ^b^the accession number for identified proteins in NCBI non-redundant (NCBInr) database; ^c^theoretical mass (kDa) and pI of identified proteins from the database; ^d^experimental mass (kDa) and pI of identified proteins; ^e^Mascot score obtained from searching the NCBInr database; ^f^Number of statistically significant matched peptides.

**Figure 3:**
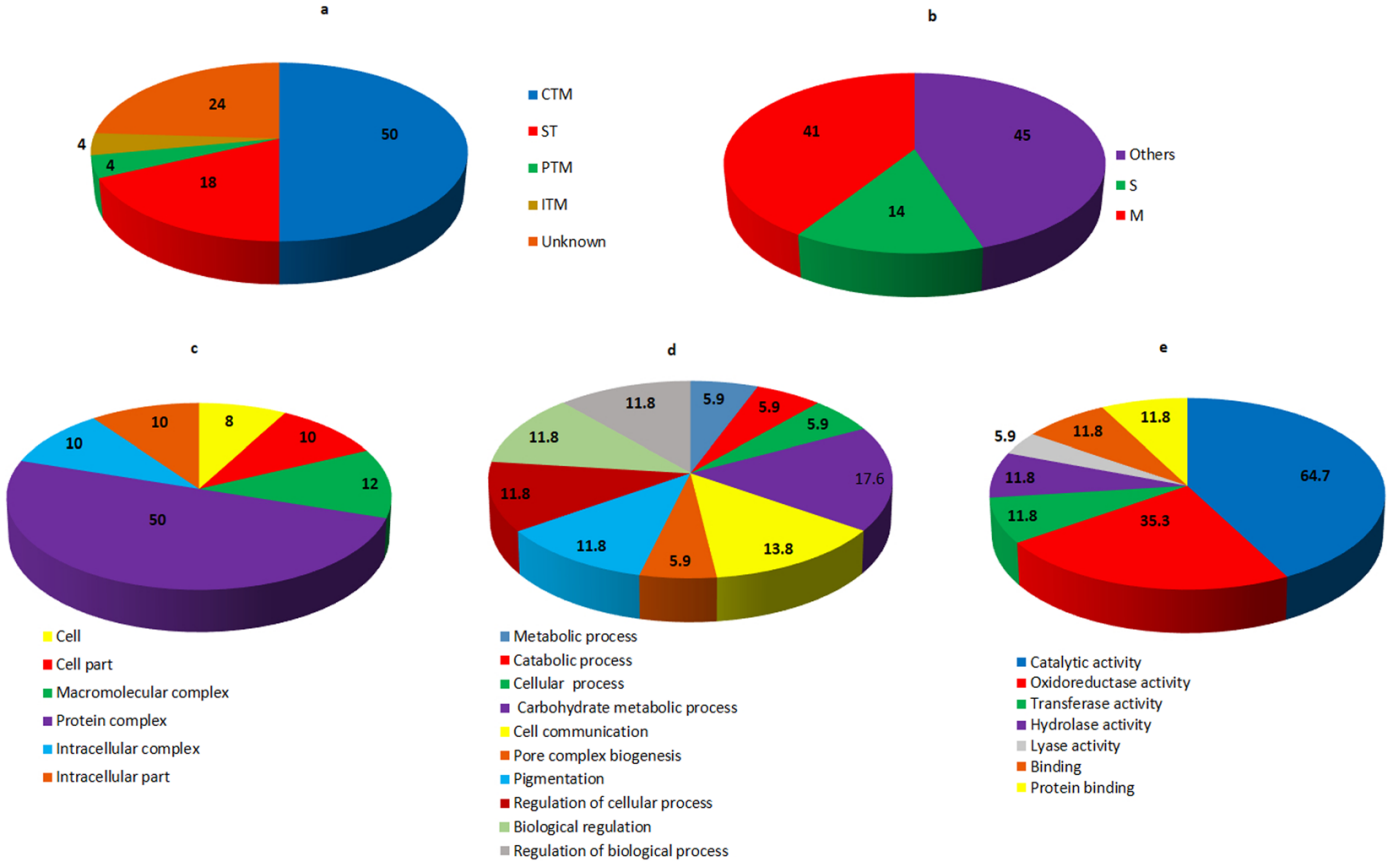
Functional analysis and sub-cellular localization of the identified proteins (**a**) Classification of identified proteins in each category of COG classification system (**b**) Sub-cellular locations of the identified proteins as determined by Target P; The analysis for cellular component (**c**), biological process (**d**) and molecular function (**e**) using Gene Ontology classification.

The functional annotation of protein spots was performed using Uniprot database. On the basis of their putative functions, the identified proteins were classified into metabolic pathways as determined by the COG classification system. About 50% of the identified proteins belong to carbohydrate transport and metabolism (CTM) classification, followed by signal transduction (ST) proteins (18%). Post- translational modification related proteins (PTM) and inorganic ion transport and metabolism related protein (ITM) comprises 4% each of the total classified proteins. 24% of the proteins belong to an unknown function classification (Fig. 3a). The sub-cellular localization of these proteins were primarily classified as any other location (45%) followed by 41% proteins localized in mitochondria and 14% proteins in the secretary pathway (Fig. 3b). For twenty one proteins varying in their abundance in TiK and TiP isolates were classified based on Gene ontology (GO) analysis into three distinct categories (cellular component, biological process and molecular function). The GO analysis for molecular function revealed that most proteins possess catalytic activity (64.7%) followed by oxidoreductase activity (35.3%) (Fig. 3e). Majority of the protein in the biological process GO analysis were involved in carbohydrate metabolic process (17.6%) and cell communication (13.8%) (Fig. 3d). While protein complexes (50%) was most dominant in cellular component category (Fig. 3c).

### Proteins involved in carbohydrate transport and metabolism may be indispensable for stronger pathogenicity

Spot1 corresponds to ATPase that is usually produced through carbohydrate metabolism^35^. ATPase is essential for providing energy for growth, development, differentiation and sporulation. Glyceraldehyde-3-phosphate dehydrogenase (GAPDH) was identified in 2 spots, spot 15 and 16 (Fig. 2; Table 2). The function of enzyme GAPDH is well known in the glycolytic cycle. However, the role of this enzyme is also implicated in various cellular processes in microbial pathogens and mammals such as DNA repair, regulation of actin filaments in microfilaments, regulation of calcium release from the ER and the nuclear export of tRNA^36–40^. Spot 2 corresponding to Glycoside hydrolases 45 (GH45) was differentially expressed in TiK isolate mycelium. β-1,3-glucanosyltransferases and Fructose-bisphosphate aldolase (FBA) were identified in spot 13 and 7, respectively (Fig. 2; Table 2).

Malate dehydrogenase (MDH) identified in spot 10, 12 and 17, was highly abundant in the mycelium of TiK isolate (Fig. 2; Table 2). Enzyme MDH is a key regulatory enzyme of tricarboxylic acid cycle that catalyses the reversible conversion of oxalacetate and malate. The product of the reaction catalysed by MDH, oxaloacetate is a precursor of oxalic acid^41^ which acts as a pathogenicity factor in *B. cinerea* and *Sclerotinia sclerotiorum*^42–47^.

### Proteins related to post translational modification and protein turnover may aid in stronger pathogenicity

HSP70 protein was highly abundant in TiK mycelium (Spot 4) (Fig. 2; Table 2). Heat shock proteins (HSPs), a highly conserved family of molecular chaperones that play a crucial role in correct folding, stabilization and post translational modification of proteins^48^. The role of some HSP 70 family proteins has been demonstrated under various abiotic stress conditions such as osmotic stress and temperature stress^49,50^.

### Protein involved in signaling pathway may contribute to stronger pathogenicity

Protein spot 6 correponding to Ste7 exhibited higher expression in mycelial protein of TiK isolate (Fig. 2; Table 2). In *S. cerevisiae*, Ste7, a MAPKK is involved in mating as well as filamentous growth and responsible for invasive growth in haploid and pseudohyphal development in diploid cells. In our laboratory, three MAP kinase homologues, namely Pmk1, Fus3 and Kpp2 have been cloned and studied using *in silico* tools, in order to define their role in fungal pathogenesis of *T. indica*^51^. In the present study, comparative proteomic analysis of mycelial proteins from isolates showing extreme virulence behaviour, TiK and TiP indicates the existence of Ste7 homolog in *T. indica* and its possible role in TiK stronger pathogenicity.

### Sequence and structure-based functional annotation

Functional annotation of putative pathogenecity/virulence related proteins is essentially required for understanding the biological processes at the molecular level. It is essential for predicting the function of protein at a systems level which is required for designing the predictive disease model^52^. There are several new methods, including hidden Markov model and neural network model–based tools available for the functional annotation of proteins which are more intelligent and efficient than classical homology search methods^53^. However, structure-based functional annotation is more reliable and much efficient for identifying protein’s biochemical or enzymatic function^54^. In present study, the identified putative pathogenecity/virulence related proteins designated as TiHsp70, TiSte7, TiFBA, TiMDH, TiGT and TiGAPDH were further subjected to comprehensive sequence and structure-based analysis: domain analysis by NCBI’s conserved domain database and Interproscan; three –dimensional protein structure prediction by RaptorX software; validation of predicted protein models by Ramachandran plot, ProQ, ProFunc, DALI server.

### TiHsp70

**Figure 4:**
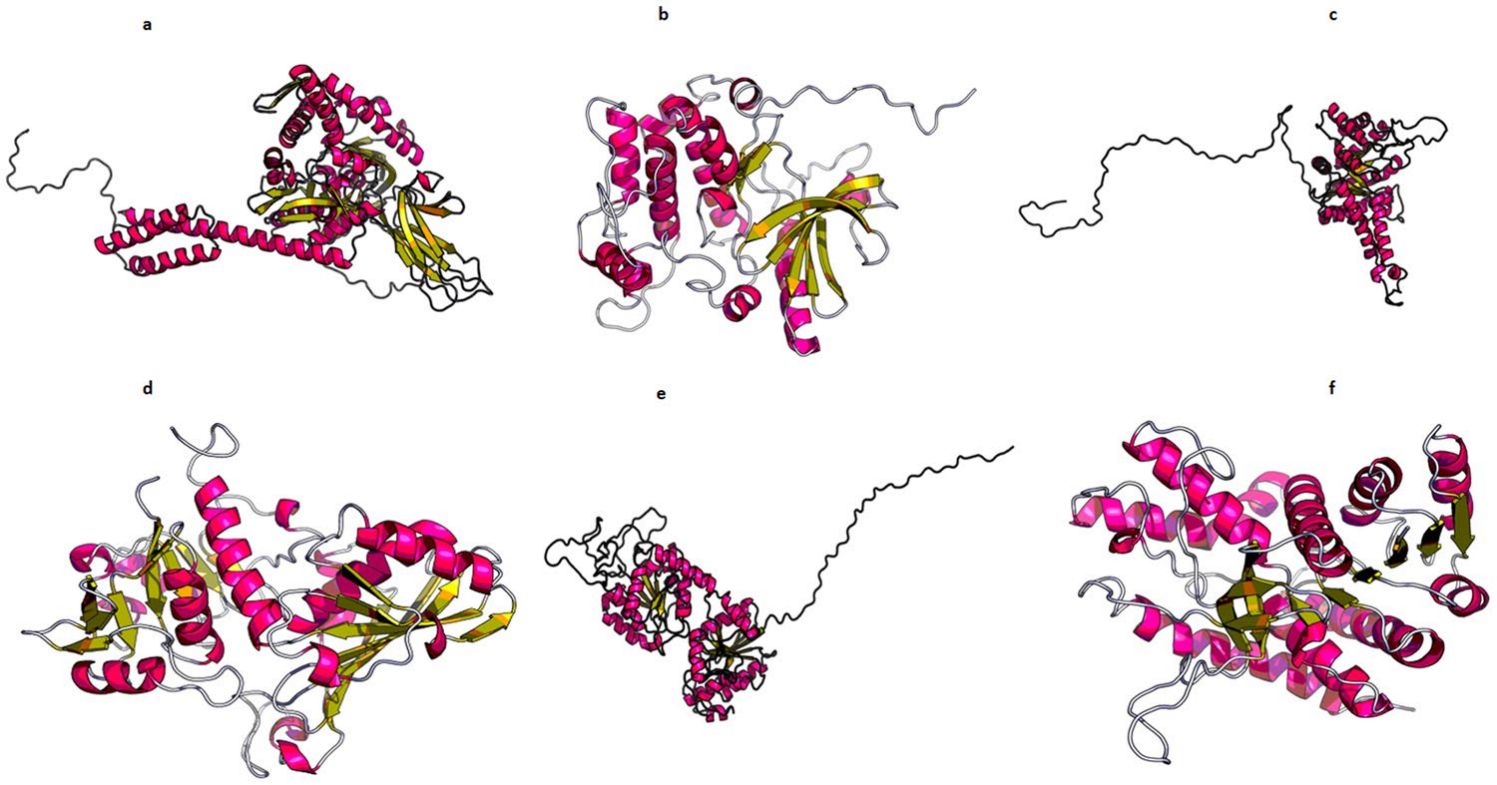
(**a**) 3-Dimensional Structure of putative pathogenicity/virulence related proteins identified from *T. indica* genome as predicted by RaptorX software^26^. Protein model for (**a**) Hsp70 (**b**) TiSte7 (**c**) TiFBA (**d**) TiGAPDH (**e**) TiGT (**f**) TiMDH.

The putative TiHsp70 sequence may act as a molecular chaperone. This protein belongs to heat shock protein HSP 70 superfamily and has two domains- the amino terminus ATPase domain and the carboxyl terminus substrate binding domain. It is ATP dependent (Table 3). The protein structures of PDB I.D. 5e84A and 5tkyA, corresponding to chaperones, as templates were used for the tertiary structure prediction. The p-value and score of 8.83e-15 and 686, respectively, were estimated for the predicted 3-D model as shown in Fig. 4a.

**Table 3:** Sequence and structural motifs present in the putative pathogenecity - related protein/virulence factors identified in mycelial proteome of *T. indica*.

Sequence	Sequence domain	Structural motifs
TiHsp70	HSP 70 superfamily	Asp126-Lys128; Ala222-Leu224
TiSte7	Serine/Threonine protein kinase	Thr354-Val356; Phe613- Arg615
TiFBA	FTBP_aldolase_IIA superfamily	Ile226-Val228; Leu311-Thr313; Gly325- Ser327
TiGAPDH	Gp_dh_N superfamily	Gly114- Ala116; Gly226- Leu228
TiGT	Glycosyltransferase_GTB_type superfamily	Glu306- Asp309
TiMDH	Malate Dehydrogenase superfamily	Gly8-Ala10; Lys221-Gly223; Lys136-Tyr139

**Figure 5:**
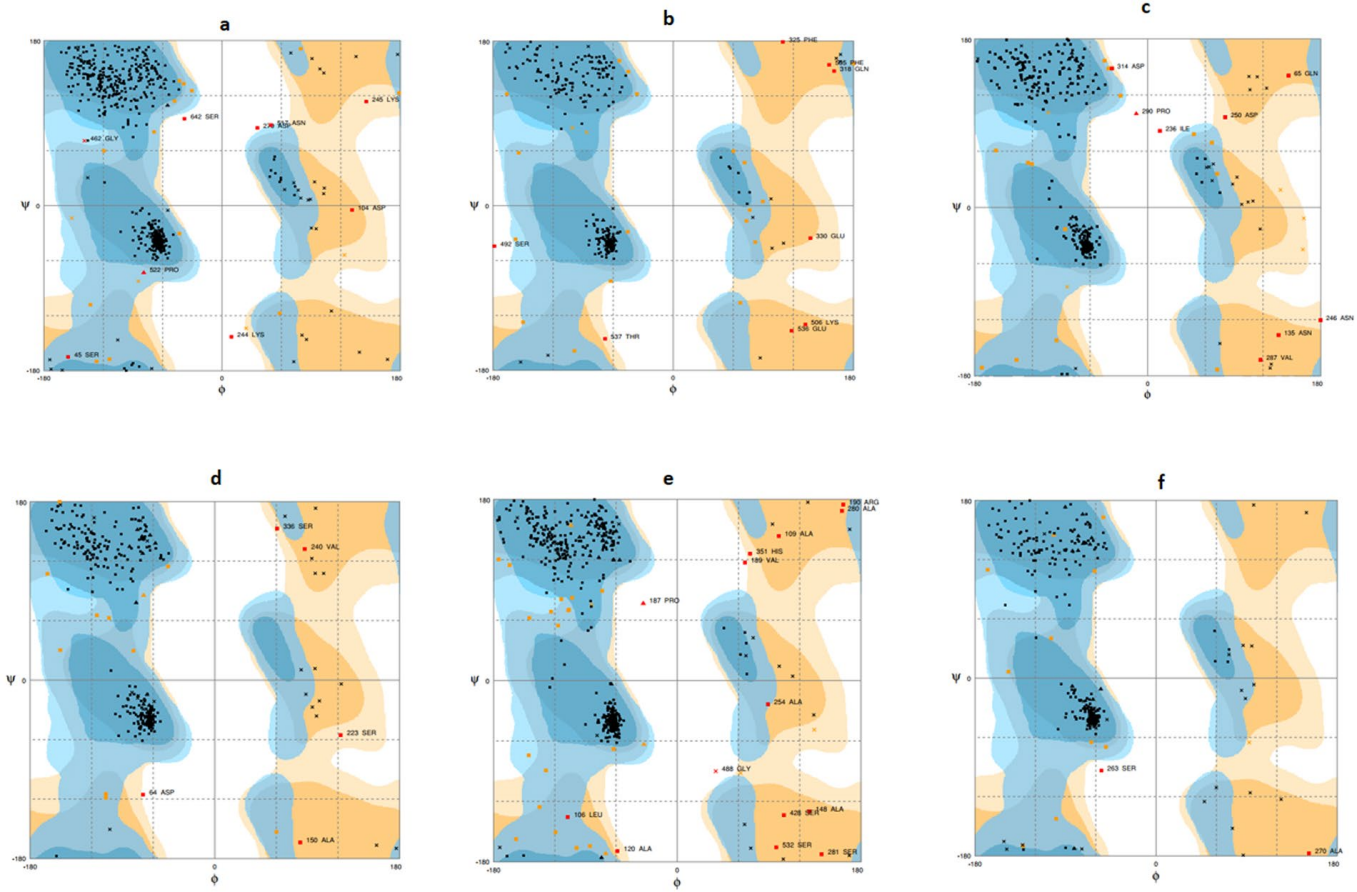
Ramachandran’s plot of 3-Dimensional protein models predicted by RaptorX software^26^ for putative pathogenicity/virulence related proteins (**a**) Hsp70 (**b**) TiSte7 (**c**) TiFBA (**d**) TiGAPDH (**e**) TiGT (**f**) TiMDH.

The statistical analysis for our model suggested that 96.0%, 2.6%, 1.4% of the residues in derived curcin model were in the favoured region, allowed region and outlier region, respectively (Fig. 5a; Table 4). Hence, around 98.6% of the residues are placed into the favoured and allowed categories, indicating the prediction of a high quality protein model (Table 4). The RMSD of the model with respect to the template (PDB code: 5e84A) is 0.49 A°, indicating similar functionality (Table 4). Moreover, ProFunc predicted HSP70_3 protein and heat shock protein Hsp70 conserved motifs showing a close resemblance with that of Hsp70 protein and Asp126-Lys128 and Ala222-Leu224 were identified as structural motifs (Table 3). We obtained the similar results on DALI server which also identified its significant similarity with Hsp70 chaperone (Z score = 68.6) (Table 4). All these analyses strongly suggest that TiHsp70 may acts as a molecular chaperone.

**Table 4:** Validation report of the protein models predicted by RaptorX software^26^.

Sequence	ProQ server		DALI server	Template	RMSD with template (A°)	Ramachandran plot
	LG score	MaxSub	Z score			Residues in favored and allowed regions (%)
TiHsp70	4.722	0.367	68.6	5e84A	0.49	98.6
TiSte7	4.292	0.44	43.6	3ornA	0.67	98.6
TiFBA	3.82	0.267	51.4	1c1f:A	0.16	98.1
TiGAPDH	5.969	0.511	56.5	4o59O	0.39	98.5
TiGT	3.161	0.242	37.2	2bisA	1.49	97.4
TiMDH	7.019	0.617	54.2	2dfdA	0.42	99.4

### TiSte7

Domain analysis results revealed that putative TiSte7 sequence possess catalytic domains of serine/threonine-specific and tyrosine-specific protein kinases (Table 3), capable of catalyzing the transfer of the γ-phosphoryl group from ATP to serine/threonine (ST) or tyrosine residues on the protein substrates. InterProScan results showed that TiSte7 sequence possesses a protein kinase domain with a catalytic core which is common to both serine/threonine and tyrosine protein kinases (Table 3). Based on these observations, it is suggested that TiSte7 may function as dual specificity MAP kinase kinase. RaptorX used protein structures of PDB I.D. 3ornA, 3zlsA, 3vn9A and 3eqcA, corresponding to mitogen-activated protein kinase, as templates for the tertiary structure prediction. p-value and score of 3.96e-10 and 278, respectively, were estimated for the predicted 3-D model as shown in Fig. 4b.

The statistical analysis for our model suggested that 96.0%, 2.6%, 1.4% of the residues in derived curcin model were in the favoured region, allowed region and outlier region, respectively (Fig. 5b; Table 4). Hence, around 98.6% of the residues were placed into the favored and allowed categories, indicating the prediction of a high quality protein model (Table 4). The RMSD of the model with respect to template was 0.67 A°, which is quite good, indicating functional similarity (Table 4). ProFunc analysis identified Serine/Threonine Protein kinase and T-X-Y motif. The structural motifs were identified as Thr354-Val356 and a Phe613- Arg615 (Table 3). DALI results showed a significant similarity with dual specificity mitogen-activated protein kinase with a maximum Z score = 43.6 (Table 4). These results also suggested a probable Serine/Threonine Protein kinase activity of TiSte7.

### TiFBA

Domains of putative TiFBA sequence is conserved with FTBP_aldolase_IIA superfamily that includes fructose-1, 6-bisphosphate (FBP) aldolase (Table 3). InterProScan result also suggested that this sequence contains FBA_II domain and has TIM beta/alpha barrel characteristic of aldolases (Table 3). RaptorX used protein structures with PDB I.D. 1c1f:A and 1dos:A as templates for the prediction of 3-D model. Template 1dos:A is Fructose-bisphosphate aldolase from *Escherichia coli* and 1c1f:A is sugar binding protein of. score for the predicted model were 7.31e-10 and 368, respectively. The 3-D structure as predicted by RaptorX software is shown in Fig. 4c.

Ramachandran plot for the predicted 3-D protein structure showed that 93.5% and 4.6% of the residues are in the favoured and allowed region 1.9% of the residues are in the outlier region. So, 98.1% of the residues were in the favored and allowed categories (Fig. 4c; Table 4), indicating the prediction of a high quality protein model. The structure of TiFBA showed a close resemblance with the template, Fructose-bisphosphate aldolase from *Escherichia coli* with an RMSD of 0.16 A° (Table 4). Functional motif search resulted in the identification of FruBisAldo_II_A and Fructose-bisphosphate aldolase class-II conserved motifs in the TiFBA. Profunc server identified Ile226-Val228, Leu311-Thr313 and Gly325- Ser327 as structurally significant motifs (Table 3). DALI search results were consistent with ProFunc finding and showed a significant similarity with the fructose-bisphosphate aldolase II (Z score = 51.4) (Table 4). These findings clearly indicated the possible fructose-bisphosphate aldolase activity of TiFBA.

### TiGAPDH

Conserved domain analysis showed that it belongs to Gp_dh_N superfamily that has Glyceraldehyde-3-phosphate dehydrogenase, containing N terminal NAD-binding domain and C terminal catalytic domain (Table 3). Interproscan results suggested that TiGAPDH sequence belongs to Glyceraldehyde/Erythrose phosphate dehydrogenase family possessing two conserved functional domains, a highly conserved catalytic domain and an NAD-binding domain. Protein Structure with PDB I.D. 4o59O, 4iq8A, 4k9dA, 3e5rA and 2vynD were used as templates for 3-D structure prediction. All the templates correspond to Glyceraldehyde-3-phosphate dehydrogenase. The predicted 3-D model had a p-value and score of 4.78e-15 and 353, respectively. This indicated the prediction of high quality 3-D model as shown in Fig. 4d.

The statistical analysis of Ramachandran plot for the predicted 3-D model suggested that 95.3% of the residues are in the favoured region. Residues in the allowed region are 3.2% while 1.5% of the residues are in the outlier region. In all, 98.5% of the residues were placed into the favored and allowed categories (Fig. 5d; Table 4). This showed that the 3-D model predicted is of high quality with respect to protein folding. The structure of TiGAPDH showed a profound similarity with the Glyceraldehyde-3-phosphate dehydrogenase from *S. cerevisiae* with RMSD of 0.39 A° (Table 4). Functional analysis using ProFunc database scan revealed the presence GAPDH-I signature in the TiGAPDH with structural motifs Gly114- Ala116 and Gly226- Leu228 (Table 3). DALI results also showed similarity with glyceraldehyde-3-phosphate dehydrogenase with Z score of 56.5 (Table 4). All these systematic analyses precisely suggested that TiGAPDH may have dehydrogenase-like activity and may be essential for pathogen survival.

### TiGT

Domain analysis suggested the similarity with Glycosyltransferase_ GTB_ type superfamily that has domain for glycosyl transferase activity (Table 3). InterProScan result also suggested that the TiGT sequence possesses a motif that is involved in glycosyl transferase activity. Protein Structure with PDB I.D. 2bisA, 5d00A, 3mboA, 3okcA and 4xsoA, corresponding to transferase, were used as templates. The predicted protein structure had a p-value and score of 2.22e-07 and 288, respectively. The predicted 3-D protein structure is given in Fig. 4e.

For Ramachandran plot, the statistical analysis of the predicted protein model showed that 92.9% of the residues are in the favoured region. Residues in the allowed region are 4.5% while 2.6% of the residues are in the outlier region. In all, 97.4% of the residues were placed into the favored and allowed categories (Fig. 5e; Table 4). These results indicated that a good quality protein model was predicted by RaptorX. The RMSD of the model with reference to the template (PDB code: 2bisA) is 1.49 A° (Table 4) which indicates the functional similarity. Functional anaylsis revealed the presence of Glycosyl transferase like conserved motif in TiGT with Glu306- Asp309 as structurally significant motif (Table 3). These results were consistent with DALI finding that showed similarity with glycosyl transferase (Z score 37.2) (Table 4). The results of sequence and structure based functional annotation suggested that TiGT possess glycosyl transferase activity.

### TiMDH

CDD result suggested that this protein sequence has a malate dehydrogenase activity with NAD binding site and substrate binding site (Table 3). This is consistent with InterProScan results that also suggested the malate dehydrogenase activity. The templates utilized by RaptorX to predict 3-D structure of TiMDH sequence according to their PDB I.D. are 2dfdA and 1sevA, corresponding to Malate dehydrogenase from human and glyoxysomal malate dehydrogenase, respectively. The predicted structure with a p-value and score of 1.52e-08 and 329, respectively indicated that model is of high quality. The 3-D structure predicted by RaptorX is shown in Fig. 4e.

The statistical analysis of Ramachandran plot for our model suggested that 96.1%, 3.3%, 0.6% of the residues in derived curcin model were in the favoured region, allowed region and outlier region, respectively. Thus, altogether 99.4% of the residues were placed into the favored and allowed categories; which indicates that the model structure derived from RaptorX was of higher quality in terms of protein folding (Fig. 5e; Table 4). The RMSD of the model with respect to the template (PDB code: 2dfdA) is 0.42 A°, indicating similar function (Table 4). Furthermore, ProFunc predicted lactate/malate dehydrogenase, alpha/beta C-terminal conserved domain and malate dehydrogenase active site signature motif showing a close resemblance with that of malate dehydrogenase protein. Gly8-Ala10, Lys221-Gly223 and Lys136-Tyr139 were identified as structural motifs (Table 3). We obtained the similar results on DALI server which also identified its significant similarity with malate dehydrogenase protein (Z score = 54.2) (Table 4). All the analyses strongly suggest that TiMDH may probably be malate dehydrogenase.

### Protein-Protein interaction network analysis of putative pathogenicity/virulence related proteins

**Figure 6:**
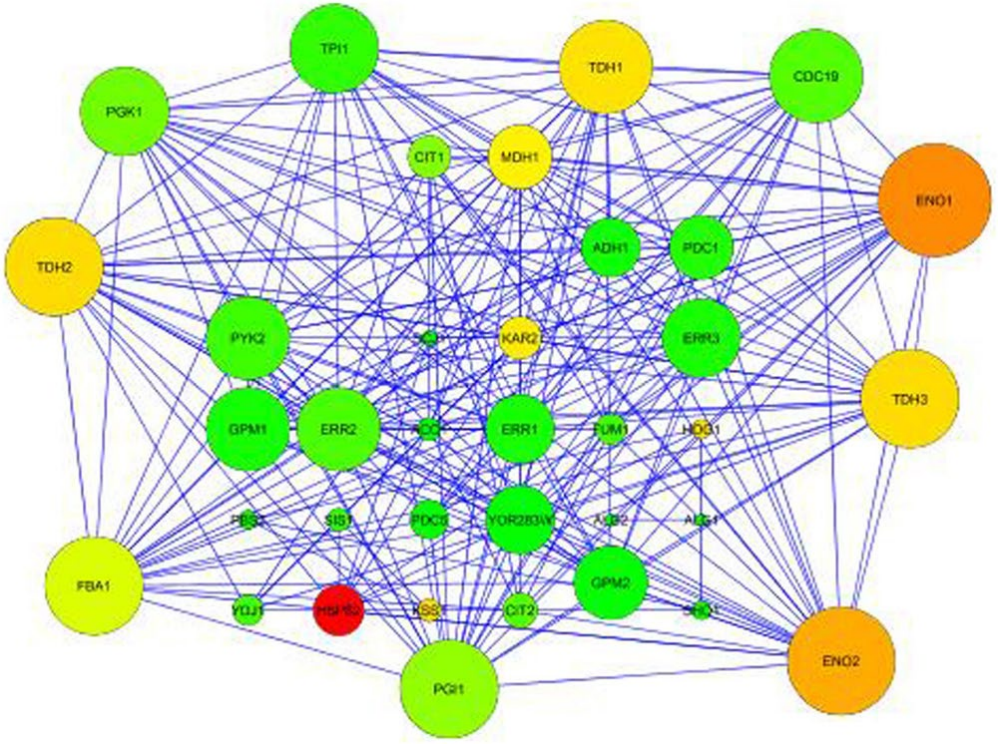
Extended protein-protein interaction (PPI) network constructed by STRING version 10.5 (Confidence score of 0.7) and visualised by Cytoscape version 3.5.1.

We have constructed an extended PPI network using putative pathogenicity/virulence related proteins from TiK genome through STRING database based on a high confidence score of 0.7. This database provides information about both experimental and predicted interactions from different sources based on their neighborhood, cooccurrence, gene fusions, co-expression, experiments and scientific literature. The constructed network implies interactions that only with high level of confidence score were taken from the database and considered as valid links. The obtained PPI network from STRING database was visualized by Cytoscape (Fig. 6). The network was analyzed topologically based on the network parameters such as node degree and betweenness centrality using Network Analyzer (Table 5). Each gene/protein in a given network is represented as a node whereas the interactions present between the nodes are defined as edges. The degree showed the number of edges linked to any node. The nodes having high degree may know as the hub proteins possessing key biological functions. The betweenness centrality represents the importance of the node, which was based on the number of shortest paths that is passing through each node. The network was visualized and analyzed based on these parameters to map the node degree as the node size and betweenness as the node color in the visualize parameter of the Network Analyzer. Nodes with high degree were displayed as a big circle and these nodes were considered as the hubs. The nodes TDH1, TDH2, TDH3 (isoforms of Glyceraldehyde-3-phosphate dehydrogenase), FBA1 (fructose-1, 6-bisphosphate aldolase), ENO1, ENO2 (isoforms of enolase), PGI (phosphoglucose isomerase), TPI (Triose phosphate isomerase) are found as hub nodes, which can be utilized as a molecular targets for development of the disease prevention and management strategies against Karnal bunt of wheat.

**Table 5:** Topological parameters used for analysis of extended protein-protein interaction (PPI) network obtained from STRING version 10.5 through Network analyzer.

Node	36	CPL	1.954
Edge	224	ND	5
CC	2	MENP	0
ANN	12.444	IN	0
SP	1124	NR	1

CC, connected component; ANN, average number of neighbors; SP, shortest path; CPL, characteristics path length; ND, network diameter; MENP, multi-edge node pair; IN, isolated node; NR, network radius.

### GC- MS based metabolic profiling of *T. indica* isolates with differing virulence behaviour

The chromatographic data of the compounds identified by GC-MS in two *T. indica* isolates are given in Tables 6 and 7. Interestingly, among saturated fatty acids, oxalic acid was exclusively identified from mycelia of highly virulent TiK isolate (Table 6). Oxalic acid is well characterized pathogenicity factor in plant pathogenic fungi, including *B. cinerea* and *S. sclerotium*^42–47^. It is synthesized from oxaloacetate which in turn is synthesized from malate in the reaction catalyzed by enzyme malate dehydrogenase (MDH). In the present study, enzyme MDH has been identified from the proteome of mycelial protein from TiK isolate. It was further identified from *de novo* assembled *T. indica* genome sequence and validated through sequence and structure based functional analysis. The present study suggests the role of oxalic acid as pathogenicity factor in KB pathogen, *T. indica*. The levels of steric acid and palmitic acid were higher in TiK samples, indicating more active fatty acid synthesis machinery in highly virulent TiK isolate. Among, unsaturated fatty acids, oleic acid level was higher in TiK sample (Table 6).

**Table 6:** Chromatographic data of the identified compounds from 70% methanol extract of highly virulent isolate TiK mycelia analyzed using GC-MS.

Identified compounds	RT	Area%	Molecular Formula	Molecular Weight
**Alcohols**				
Nonen-1-ol	22.83	0.48	C9H18O	142
**Saturated fatty acids**				
Oxalic acid	26.24	1.57	C16H30O4	286
Stearic acid	21.17	15.76	C18H36O2	284
Palmitic acid	21.82	27.43	C16H32O2	256
**Unsaturated fatty acids**				
Petroselinic acid	22.89	11.93	C19H36O2	296
Oleic acid	23.53	50.45	C19H36O2	296

**Table 7:** Chromatographic data of the identified compounds from 70% methanol extract of low virulent isolate TiP mycelia analyzed using GC-MS.

Identified compounds	RT	Area%	Molecular Formula	Molecular Weight
**Saturated fatty acids**				
Palmitic acid	21.18	15.54	C16H32O2	256
Stearic acid	23.12	8.14	C18H36O2	284
**Unsaturated fatty acids**				
Oleic acid	22.93	15.76	C19H36O2	296
Linoleic acid	23.75	18.4	C18H32O2	279

## Discussion

Selection and use of *T. indica* resistant wheat varieties is a key to successful KB disease management. The present study showed that the host-pathogen reactions were quite consistent, regardless of the environmental conditions. Various *T. indica* isolates exhibited differential reactions on a set of differential host genotypes. It also confirms the earlier studies regarding the existence of pathogenic variability in *T. indica*. It is revealed that the high aggressive type (KB-HAg) was present in TiK while the low aggressive type in TiP isolate.

Comparative proteomic analysis of mycelial proteins from TiK and TiP isolates revealed that the proteins associated with carbohydrate metabolism, namely Glyceraldehyde-3-phosphate dehydrogenase, Malate dehydrogenase, Fructose-1,6-bisphosphate aldolase and Glucanosyltransferase showed highest percentage (50%) in the mycelium of virulent TiK isolate. Higher expression of proteins associated with carbohydrate metabolism in mycelia of TiK than TiP is consistent with the fact that TiK has higher growth rate than TiP isolate. The fast growing TiK mycelium requires more energy than TiP.

GAPDH was highly abundant in the TiK mycelium. GAPDH serve as virulence factor in several pathogenic fungi, including *Candida albicans*, *Trichomonas vaginalis*, *Phycomyces Blakesleeanus*, *Paracoccidioides brasiliensis* and *Botrytis cinerea*^36–40^. Apart from cytosol, a GAPDH is also present on the surface of pathogenic fungi where it binds to host’s fibronectin. This facilitates pathogen to localize at intracellular and extracellular matrix environment and aid in invasion and colonization of the host tissue. In the present study, spot 16 identified as GAPDH was uniquely present in the mycelia of TiK isolate, indicating that the oxidative metabolism in the highly virulent isolate might be much more active than that of the low virulent isolate. It may also be hypothesized the presence of surface GAPDH in virulent *T. indica* isolate and its role in facilitating fungal invasion and colonization of host wheat tissues. This suggested a putative role of enzyme GAPDH as virulence factor in *T. indica*. In TiK, FBA that catalyzes the aldol cleavage of fructose bisphosphate, was highly abundant. In *Candida albicans*, a mutation in FBA gene of perturbs the fungal growth^55^, FBA present on the surface serve as an important virulence factor in *Paracoccidioides*, by involving in the process of fungal adhesion, invasion and colonization. The FBA protein was also found to be upregulated in *Fusarium oxysporum* race 4 displaying stronger virulence^56^. FBA may also be present on the surface of TiK isolate that might play crucial role in *T. indica* adhesion and invasion and act as important virulence factor.

The role of β-1,3-glucanosyltransferases (spot 13) have been implicated in the fungal cell wall biosynthesis, morphogenesis and virulence of human pathogenic fungi such as *Candida albicans* and *Aspergillus fumigates*^57^. Caracuel *et al*.^58^ examined the role of putative β-1,3-glucanosyltransferase encoding gas1 gene of vascular wilt pathogen, *Fusarium oxysporum*. They observed that gas1 deletion mutants of *F. oxysporum* exhibited a significantly lower growth rates, increased resistance to cell wall–degrading enzymes. Li *et al*.^34^ studied the significance of β-1,3-glucanosyltransferase in virulence of *F. oxysporum f. sp.conglutinans* on cabbage plants. The *F. oxysporum f. sp.conglutinans* gas1 deletion mutants showed restricted growth and virulence compared to wild type isolates. These results were consistent with the comparative proteomic analysis of two races of *F. oxysporum f. sp. conglutinans* differing in pathogenicity as glucanosyltransferase showed higher expression in highly pathogenic Race R2. Our comparative proteomic analysis of TiK and TiP isolate also exhibited higher expression of glucanosyltransferase protein in isolate showing stronger pathogenicity. This indicates the essential role of glucanosyltransferase in KB pathogenesis on wheat.

For plant pathogenic fungi, the plant cell wall is the main barrier that is required to breach for colonizing the host plant tissues. They produce an arsenal of cell wall degrading enzymes that are responsible for degrading cellulose. Among them, Glycoside hydrolases of family GH45 (endo-β-1,4-glucanase activity) are of prime importance that cleaves the internal glycosidic bond leaving β-1,4 glucans from the straight chain polymer of glucose, cellulose. The endo-β-1,4-glucanase CelA in *Clavibacter michiganensis* subsp. *michiganensis* is a pathogenicity determinant that is necessary for inducing the bacterial wilt in tomato^59^. So far, no information is available regarding the relationship between the endocellulase and *T. indica* pathogenicity. In this study, Spot 2 corresponding to GH45 endoglucanases was differentially expressed in TiK isolate mycelium may be responsible for its stronger pathogenicity (Fig. 2; Table 2).

Under biotic stress, both host and pathogen are exposed to various extreme detrimental stress stimuli such as cell wall degrading enzymes, acidic pH and reactive oxygen species. In both host and pathogen, expression of various HSPs are up-regulated with pathogen HSPs being responsible for infection of the host plant and host HSPs in turn provide defense against the pathogen invasion^60–65^. Studies led by Yi *et al*.^66^ showed the significance of HSP70 family proteins LHS1, KAR2 of *Magnaporthe oryzae* in fungal pathogenicity. The present study suggested that TiK isolate mycelia HSP70 protein abundance may enhance mycelial tolerance to extreme stress condition (viz. acidic pH, cell wall degrading enzymes and reactive oxygen species) that are generated during pathogen invasion of host tissues and ultimately contribute to fungal virulence.

Malate dehydrogenase abundance was high in TiK mycelium (spot 10, 12 and 17). Fernandez-Acero *et al*.^7^ have suggested the role of MDH as a pathogenicity factor in *B. cinerea* as oxalic acid secretion generates an acidic environment that create an appropriate ecological niche for the fungal pathogenic activities such as secretion of several virulence factors like cell wall degrading enzymes and phytotoxins^67,68^. There is a correlation between oxalic acid biosynthetic capability and *S. sclerotiorum* virulence as mutants lacking oxalic acid biosynthesis machinery were found to be non-pathogenic while revertant strains that regained their oxalic acid synthesis ability exhibited normal virulence^43^. Likewise, MDH higher expression in virulent TiK isolate mycelia suggested the relation of oxalate synthesis with enhanced fungal virulence. Further, oxalic acid might play a crucial role in invasion of host plant cell by lowering the pH and providing suitable acidic environment for the activity of arsenal of cell wall degrading enzymes. GC-MS based metabolic profiling identified oxalic acid from TiK mycelium, substantiating the role of oxalic acid as potential pathogenicity factor in *T. indica*.

## Conclusion

Despite quarantine significance of *T. indica*, there is a little knowledge regarding the molecular mechanisms of pathogenesis employed by this important fungus to cause disease. Moreover, all the methods used to manage the disease have proven futile. In order to develop an effective disease management strategy, it is essential to identify pathogenicity or virulence related proteins. However, so far not much information about pathogenic determinants of this economically important fungus is available and certainly not using Hi-throughput proteomics and metabolic tools and platforms. In the present study, we report the first proteome map and comprehensive comparative proteomic analysis of *T. indica* isolates differing in their virulence/aggressiveness for identification of putative pathogenicity or virulence related proteins expressed in the highly virulent isolate. The identified pathogenecity/virulence related proteins play crucial role in stress response, degradation of host cell wall, adhesion, penetration, invasion, colonization, activation of signal transduction pathway and morphogenesis. Potential pathogenecity/virulence related proteins were further complemented with *T. indica* genome sequence from hybrid genome assembly, resulting in identification of orthologs of several candidate pathogenecity/virulence related proteins which were annotated through both sequence and structure - based functional analysis. Further, GC-MS based metabolic profiling of *T. indica* isolates varying in virulence behaviour validates the role of oxalic acid as potential pathogenicity factor in *T. indica*. The identified pathogenecity/virulence related proteins may serve as potential biomarkers that would have utility for screening KB resistant wheat cultivars, designing target specific fungicides and development of specific, sensitive and rapid field level on - site KB diagnostics.

## Electronic supplementary material

Supplementary file

## References

[1] Mitra M (1931). A new bunt of wheat in India. Annual Applied Biology.

[2] Mehdi V, Joshi LM, Abrol YP (1973). Studies on chapatti quality. VI. Effect of wheat grains with bunts on the quality of ‘chapatties’. Bulletin of Grain Technology.

[3] Jones DR (2007). A reappraisal of the current status of *Tilletia indica* as an important quarantine pest for Europe. European Journal of Plant Pathology.

[4] Rush CM (2005). Status of Karnal Bunt of Wheat in the United States 1996 to 2004. Plant Disease.

[5] Yoder OC, Turgeon BG (2001). Fungal Genomics and Pathogenicity. Current Opinions in Plant Biolog.

[6] Fernández-Acero FJ (2007). Proteomic analysis of phytopathogenic fungus *Botrytis cinerea* as a potential tool for identifying pathogenicity factors, therapeutic targets and for basic research. Archives of Microbiology.

[7] Fernández-Acero FJ (2006). Two-dimensional electrophoresis protein profile of the phytopathogenic fungus *Botrytis cinerea*. Proteomics.

[8] El-Bebany AF, Rampitsch C, Daayf F (2010). Proteomic analysis of the phytopathogenic soilborne fungus Verticillium dahliae reveals differential protein expression in isolates that differ in aggressiveness. Proteomics..

[9] Kumar A (2017). Draft genome sequence of Karnal bunt pathogen (Tilletia indica) of wheat provides insights into the pathogenic mechanisms of quarantined fungus. PLOS ONE.

[10] Parker D (2009). Metabolomic analysis reveals a common pattern of metabolic re-programming during invasion of three host plant species by Magnaporthe grisea. The Plant Journal.

[11] Allwood JW, Ellis DI, Heald JK, Goodacre R, Mur LA (2006). Metabolomic approaches reveal that phosphatidic and phosphatidyl glycerol phospholipids are major discriminatory non-polar metabolites in responses by Brachypodium distachyon to challenge by *Magnaporthe grisea*. Plant Journal.

[12] Warham, E. J. Studies on Kamal Bunt of Wheat. Ph.D. thesis. University of Wales. Aberyshth, UK (1987).

[13] Tandon, J. P. &, Sethi, A. P. Wheat Production Technology. Directorate of Wheat Research, Karnal. pp. **10** (1991).

[14] Zadocks JC, Chang TT, Konzak CF (1974). A decimal code for the growth stage of cereals. Weed Research.

[15] Aujla SS, Sharma I, Singh BB (1987). Physiologic specialization of Karnal bunt of wheat. Indian Phytopathology.

[16] Aujla SS, Sharma I, Singh BB (1989). Rating scale for identifying wheat varieties resistant to *Neovossia indica* (Mitra) Mundkur. Indian Phytopathology.

[17] Bradford MM (1976). A rapid and sensitive method for the quantitation of microgram quantities of protein utilizing the principle of protein-dye binding. Analytical Biochemistry.

[18] Fragner D, Zomorrodi M, Kues U, Majcherczyk A (2009). Optimized protocol for the 2-DE of extracellular proteins from higher basidiomycetes inhabiting lignocellulose. Electrophoresis.

[19] Emanuelsson O, Nielsen H, Brunak S, Heijne GV (2000). Predicting subcellular localization of proteins based on their N-terminal amino acid sequence. Journal of Molecular Biology.

[20] Quevillon E (2005). InterProScan: protein domains identifier. Nucleic Acids Research.

[21] Marchler-Bauer A (2011). CDD: a conserved domain database for the functional annotation of proteins. Nucleic Acids Research..

[22] de Castro E (2006). ScanProsite: detection of PROSITE signature matches and ProRule-associated functional and structural residues in proteins. Nucleic Acids Research.

[23] Letunic I, Doerks T, Bork P (2012). SMART 7: recent updates to the protein domain annotation resource. Nucleic Acids Research.

[24] Thomas PD (2003). PANTHER: a library of protein families and subfamilies indexed by function. Genome Research.

[25] Orengo CA (1997). CATH—a hierarchic classification of protein domain structures. Structure.

[26] Kallberg M (2012). Template-based protein structure modeling using the RaptorX web server. Nature Protocols.

[27] Lovell SC (2002). Structure validation by Calpha geometry: phi,psi and Cbeta deviation. Proteins: Structure, Function & Genetics.

[28] Wallner B, Elofsson A (2003). Can correct protein models be identified?. Protein Science.

[29] Holm L, Rosenström P (2010). Dali server: conservation mapping in 3D. Nucleic Acids Research.

[30] Laskowski RA, Watson JD, Thornton JM (2005). ProFunc: a server for predicting protein function from 3D structure. Nucleic Acids Research.

[31] Szklarczyk D (2017). The STRING database in 2017: quality-controlled protein-protein association networks, made broadly accessible. Nucleic Acids Res..

[32] Shannon P (2003). Cytoscape: a software Environment for integrated models of biomolecular interaction networks. Genome Research.

[33] Oh TJ (2014). NMR and GC-MS Based Metabolic Profiling and Free-Radical Scavenging Activities of *Cordyceps pruinosa* Mycelia Cultivated under Different Media and Light Conditions. PLOS ONE.

[34] Li E (2015). Comparative Proteomics Analyses of Two Races of *Fusarium oxysporum f. sp. conglutinans* that Differ in Pathogenicity. Scientific Reports.

[35] Wang X (2009). Comparative proteomic analysis of differentially expressed proteins in shoots of *Salicornia europaea* under different salinity. Journal of Proteome Research.

[36] Hernandez R, Nombela C, Diez-Orejas R, Gil C (2004). Two-dimensional reference map of *Candida albicans* hyphal forms. Proteomics.

[37] Alderete JF, Millsap KW, Lehker MW, Benchimol M (2001). Enzymes on microbial pathogens and *Trichomonas vaginalis*: molecular mimicry and functional diversity. Cellular Microbiology.

[38] Deveze-Alvarez M, Garcia-Soto J, Martinez-Cadena G (2001). Glyceraldehyde-3-phosphate dehydrogenase is negatively regulated by ADP-ribosylation in the fungus *Phycomyces blakesleeanus*. Microbiology.

[39] Pancholi V, Chhatwal GS (2003). Housekeeping enzymes as virulence factors for pathogens. International Journal of Medical Microbiology.

[40] Barbosa MS (2006). Glyceraldehyde-3-phosphate dehydrogenase of *Paracoccidioides brasiliensis* is a cell surface protein involved in fungal adhesion to extracellular matrix proteins and interaction with cells. Infection and Immunity.

[41] Kubicek CP, Schreferl-Kunar G, Wohrer W, Rohr M (1988). Evidence for a Cytplasmatic Pathway of Oxalate Biosynthesis in *Aspergillus niger*. Applied Environmental Microbiology.

[42] Lyon, G. D., Goodman, B. A. & Williamson, B. *Botrytis cinerea* perturbs redox processes as an attack strategy in plants. *Botrytis*: biology, pathology and control. *In: Elad Y, Williamson B*, Tudzynski *P, Delen N, editors. (Dordrecht: Springer)* 119–141 (2004).

[43] Godoy G, Steadman JR, Dickman MB, Dam R (1990). Use of mutants to demonstrate the role of oxalic acid in pathogenicity of *Sclerotinia sclerotiorum* on *Phaseolus vulgaris*. Physiological and Molecular Plant Pathology.

[44] Dickman MB, Mitra A (1992). *Arabidopsis* as a model for studying resistance to *Sclerotinia* infection. Physiological and Molecular Plant Pathology.

[45] Rollins JA, Dickman MB (1998). Inhibition of sclerotial development in *Sclerotinia sclerotiorum* by increasing endogenous and exogenous cAMP levels. Applied Environmental Microbiology.

[46] Rollins JA, Dickman MB (2001). pH signaling in *Sclerotinia sclerotiorum*: Identification of apacC/RIMI homolog. Applied Environmental Microbiology.

[47] Cessna SG, Sears VE, Low PS, Dickman M (2000). Oxalic acid, a pathogenicity factor for *Sclerotinia sclerotiorum*, suppresses the oxidative burst of the host plant. Plant Cell.

[48] Ellis RJ, Hartl FU (1999). Principles of protein folding in the cellular environment. Current Opinion in Structural Biology.

[49] Holden DW, Kronstad JW, Leong SA (1989). Mutation in a heat-regulated hsp70 gene of *Ustilago maydis*. EMBO Journal.

[50] Salmeron-Santiago KG (2011). Response to osmotic stress and temperature of the fungus *Ustilago maydis*. Archives of Microbiology.

[51] Gupta AK, Goel A, Seneviratne JM, Joshi GK, Kumar A (2011). Molecular Cloning of MAP Kinase Genes and In silico Identification of their Downstream Transcription Factors Involved in Pathogenesis of Karnal bunt (*Tilletia indica*) of Wheat. Journal of Proteomics & Bioinformatics.

[52] Mazandu GK, Mulder NJ (2012). Function prediction and analysis of *Mycobacterium* tuberculosis hypothetical proteins. International Journal of Molecular Sciences.

[53] Rashid M, Saha S, Raghava GP (2007). Support Vector Machine based method for predicting subcellular localization of mycobacterial proteins using evolutionary information and motifs. BMC Bioinformatics.

[54] Haas BJ, Zeng Q, Pearson MD, Cuomo CA, Wortman JR (2011). Approaches to fungal genome annotation. Mycology.

[55] Rodaki A, Young T, Brown AJ (2006). Effects of depleting the essential central metabolic enzyme fructose-1, 6-bisphosphate aldolase on the growth and viability of *Candida albicans*: implications for antifungal drug target discovery. Eukaryotic Cell.

[56] Sun Y (2014). Proteomics of *Fusarium oxysporum* Race 1 and Race 4 Reveals Enzymes Involved in Carbohydrate Metabolism and Ion Transport That Might Play Important Roles in Banana *Fusarium* Wilt. PLOS ONE.

[57] Mouyna I (2000). Glycosylphosphatidylinositol – anchored glucanosyltransferases play an active role in the biosynthesis of the fungal cell wall. The Journal Biological Chemistry.

[58] Caracuel Z, Martínez-Rocha AL, Di Pietro A, Madrid MP, Roncero MI (2005). *Fusarium oxysporum* gas1 encodes a putative beta-1,3-glucanosyltransferase required for virulence on tomato plants. Molecular Plant Microbe Interaction.

[59] Jahr H, Dreier J, Meletzus D, Bahro R, Eichenlaub R (2000). The endo-beta-1,4-glucanase CelA of *Clavibacter michiganensis subsp. Michiganensis* is a pathogenicity determinant required for induction of bacterial wilt of tomato. Molecular Plant-Microbe Interaction.

[60] Liu HY, Dicksved J, Lundh T, Lindberg JE (2014). Expression of heat shock protein 27 and 72 correlates with specific commensal microbes in different regions of porcine gastrointestinal tract. American Journal of Physiology. Gastrointestinal and Liver Physiology.

[61] Buchmeier NA, Heffron F (1990). Induction of *Salmonella* stress proteins upon infection of macrophages. Science.

[62] Gahan CG, O’Mahony J, Hill C (2001). Characterization of the groESL operon in *Listeria monocytogenes*: utilization of two reporter systems (gfp and hly) for evaluating *in vivo* expression. Infection and Immunity.

[63] Monahan IM, Betts J, Banerjee DK, Butcher P (2001). Differential expression of mycobacterial proteins following phagocytosis by macrophages. Microbiology.

[64] Gaywee J, Radulovic S, Higgins JA, Azad AF (2002). Transcriptional analysis of *Rickettsia prowazekii* invasion gene homolog (invA) during host cell infection. Infection and Immunity.

[65] Schnappinger D (2003). Transcriptional adaptation of Mycobacterium tuberculosis within macrophages: insights into the phagosomal environment. The Journal of Experimental Medicine.

[66] Yi M (2009). The ER chaperone LHS1 is involved in asexual development and rice infection by the blast fungus *Magnaporthe oryzae*. Plant Cell.

[67] Wubben JP, ten Have A, van Kan JAL, Visser J (2000). Regulation of endopolygalacturonase gene expression in Botrytis cinerea by galacturonic acid, ambient pH and carbon catabolite repression. Current Genetics.

[68] Manteau S, Abouna S, Lambert B, Legendre L (2003). Differential regulation by ambient pH of putative virulence factor secretion by the phytopathogenic fungus *Botrytis cinerea*. FEMS Microbiology Ecology.

