# Supplementary material for: Integrated proteomics, genomics, metabolomics approaches reveal oxalic acid as pathogenicity factor in Tilletia indica inciting Karnal bunt disease of wheat
Source: Sci Rep. 2018 May 18;8:7826. doi: 10.1038/s41598-018-26257-z (PMC5959904; doi:10.1038/s41598-018-26257-z)
Supplement: Supplementary file 1 — Supplementary file [file 41598_2018_26257_MOESM1_ESM.docx]

**Integrated proteomics, genomics, metabolomics approaches reveal oxalic acid as pathogenicity factor in *Tilletia indica* inciting Karnal bunt disease of wheat**

**Vishakha Pandey^1^, Manoj Singh^1^, Dinesh Pandey^1^ and Anil Kumar*^1^**

Author Affiliations

1 Department of Molecular biology and Genetic Engineering, G.B. Pant University

of Agriculture and Technology , Pantnagar, Uttarakhand India.

*Corresponding author:

E-mail: [ak_gupta2k@rediffmail.com](mailto:ak_gupta2k@rediffmail.com), [anilkumar.mbge@gmail.com](mailto:anilkumar.mbge@gmail.com).


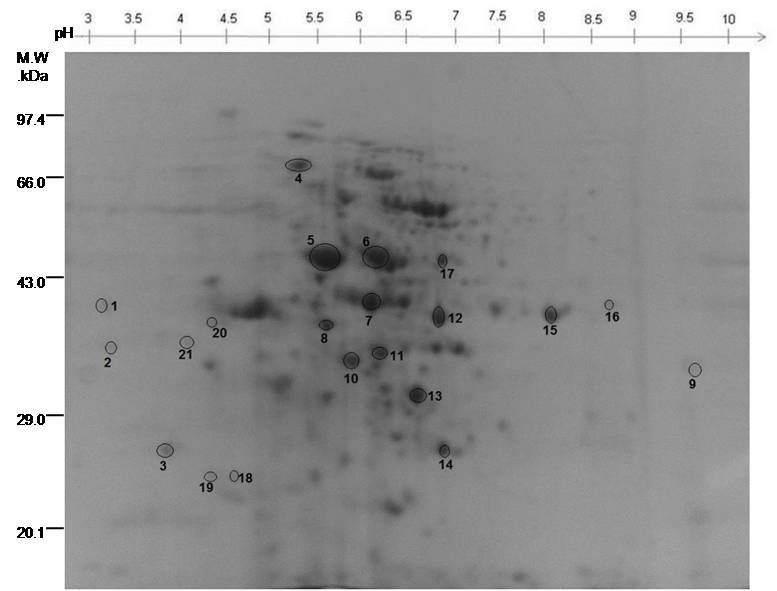


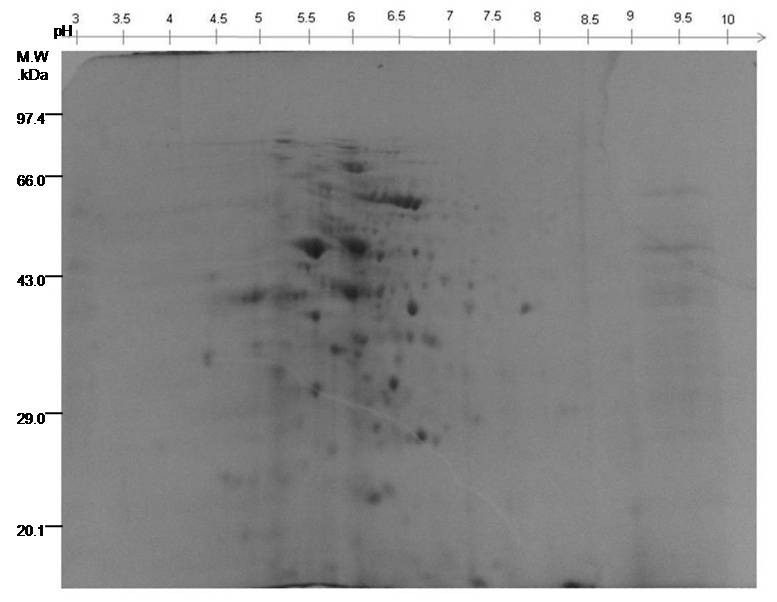


**Figure S1: Representative Coomassie Brilliant Blue (CBB)-stained two-dimensional electrophoresis (2-DE) gels of proteins extracted from mycelia of *T. indica* isolates (at 21 days of growth) showing contrasting virulence behaviour (a) 2-DE gel image of TiK, highly virulent isolate (b) TiP, low virulent isolate. The MW (kDa) and pI scales are indicated.**
